# A Visit to the Pediatrician as a Part of Comprehensive Prenatal Care?

**DOI:** 10.1007/s10995-023-03791-5

**Published:** 2023-10-21

**Authors:** Tiffany L. Mei, C. Andrew Aligne, Jessica L. VanScott

**Affiliations:** 1 Department of Obstetrics and Gynecology, Tufts Medical Center, Boston, USA; 2 Department of Pediatrics, The Hoekelman Center, Golisano Children’s Hospital, University of Rochester School of Medicine and Dentistry, 601 Elmwood Avenue Box 777, Rochester, NY 14642 USA; 3 Infectious Diseases Division, Center for Community Practice, University of Rochester School of Medicine and Dentistry, Rochester, NY USA

**Keywords:** Prenatal care, Immunization hesitancy, Disparities in maternal and child health, Breastfeeding aids, Parenting of newborn

## Abstract

**Introduction:**

The American Academy of Pediatrics and the American College of Obstetricians and Gynecologists recommend that expectant parents receive a preventive visit with a pediatrician in the prenatal period (a pediatric prenatal visit, or PPV). Discussing newborn health topics in the prenatal period tends to be more effective than immediately postpartum, and research suggests, for example, that the PPV increases timely childhood immunizations. However, only 22% of expectant parents have these visits, and there are significant disparities by race and income.

**Methods:**

A 2-min online survey with open-ended questions was emailed to 304 eligible obstetrics providers in Rochester, NY. Simple descriptive statistics and chisquare analysis were applied to survey responses. Responses were organized within the framework of knowledge, attitudes, and practices to identify barriers to guideline adherence resulting in chronic under-utilization of the PPV.

**Results:**

Ninety obstetric providers completed the survey. 66 reported awareness of the PPV, and 45 reported referring patients for a PPV. However, in open-ended questions, respondents expressed confusion between the PPV and a “meet and greet” visit with a pediatrician. Some respondents believed that the PPV is not covered by insurance, even though these visits are covered by Medicaid and marketplace insurance. Providers who had personally received one as a parent expressed positive attitudes.

**Discussion:**

These findings indicate that unfamiliarity with the PPV is one barrier to referral. Educating providers about the guideline recommendations, evidence base, and insurance coverage could overcome this barrier. Doing so could reduce disparities in utilization of the pediatric prenatal visit.

## Significance

*What’s Known on This Subject* The pediatric prenatal visit is an opportunity to deliver anticipatory guidance and to gather family information. Despite evidence indicating that pediatric prenatal visits enhance outcomes like immunization rates, few expectant parents receive this service—especially in low-income and BIPOC populations.

*What This Study Adds* Even after recent joint AAP and ACOG guidelines recommending the pediatric prenatal visit, it appears that awareness among obstetrics providers remains low. Disseminating information about existing evidence-based guidelines to patients and providers could increase PPV awareness, reduce disparities in its utilization, and improve health outcomes for mothers and children.

## Introduction

The most recent Guidelines for Perinatal Care, as established by the American Academy of Pediatrics (AAP) and the American College of Obstetricians and Gynecologists (ACOG), published in 2017, state that “sometime during the third trimester, the pregnant woman should be encouraged to meet with a newborn care provider to discuss the importance of newborn care, including the importance of vaccines and feeding” (AAP Committee on Newborn and Fetus, 2017). Establishing care with a primary care provider for the infant optimizes postpartum care for the mother and is part of establishing a medical home for a child (ACOG, 2018). The pediatric prenatal visit (PPV) allows time for expectant parents to meet with a pediatrician (or other child primary care provider) and receive anticipatory guidance on topics such as breastfeeding, childhood immunizations, circumcision, newborn screening, and general newborn care (Yogman et al., 2018). These are topics that new mothers are frequently concerned about postpartum (Kanotra et al., 2007). These visits also serve as an opportunity for pediatricians to discuss topics such as positive newborn screening, congenital anomalies, and fetal exposures regarding expectations as parents transition care to the primary pediatrician (Yogman et al., 2018). The visit is also an opportunity for the newborn’s physician to gather information about family history, parental preferences, and psychosocial and environmental concerns.

Discussing newborn health topics in the prenatal period tends to be more effective than in the immediate postpartum period, when women are undergoing acute physical, emotional, and social adjustments and may be less receptive to processing information (Fakhraei & Terrion, 2017; Moran et al., 1997). Research suggests, for example, that the PPV increases timely hepatitis B immunizations at birth and routine childhood immunizations thereafter as recommended by the CDC (Navar et al., 2007; Saitoh et al., 2013).

While 73% of pediatricians offer prenatal visits, only 22% of expectant parents will have a PPV, and there are significant disparities by race and income (Yogman et al., 2018; Martin et al., 2021; Bryant et al., 2010). Only 5% of low-income women have an appointment with a pediatric provider in the prenatal period (Berger & Rose, 1983). This is particularly important because urban, low-income families who have received a PPV experience increased breastfeeding, reduced emergency room visits, and better doctor-patient relationships (Serwint et al., 1996).

Comprehensive prenatal care including the PPV leads to better outcomes across racial groups (Sparks, 2009; Weir et al., 2011).

The PPV is covered by Medicaid, and therefore cost per se should not be a barrier. Passage of the Affordable Care Act (ACA) in 2010 has further removed financial barriers to prenatal care, and services offered in the PPV apply to all pregnancies regardless of when insurance coverage starts (HealthCare.gov, 2021). A reminder or referral from an obstetric provider is an important route to the PPV because otherwise many parents, especially first-time mothers, are not aware of the existence of these visits and have not identified a pediatrician prior to delivery, which leads to gaps in transition of care (Sprunger & Preece 1981). There has been limited research in the utilization of the PPV in decades, despite recent recommendations by the AAP and ACOG. To develop feasible solutions for overcoming the chronic under-utilization of the PPV, we sought in this pilot study to identify barriers to guideline adherence using a framework of identifying knowledge, attitudes, and practices (Cabana et al., 1999).

## Methods

We conducted a survey with open-ended questions to investigate obstetric providers’ knowledge, attitudes, and practices regarding the PPV. A list of obstetric providers was obtained for the two major health systems in Rochester, NY by contacting the department administration. This population consisted of obstetric practitioners, including obstetricians, family medicine physicians, midwives, advanced practice providers, and nurses. Physicians at all levels of training were surveyed. The one-time 2-min online survey was sent to 367 potential participants and collected over a period of 4 weeks from May 2021-June 2021. Fourteen individuals were excluded for not being obstetric providers, 37 were excluded because they did not directly provide obstetric care but were associated with obstetrics (e.g. practice managers), nine were excluded for failed email delivery, and three were excluded for having a prolonged out of office message. There was a total of 304 providers eligible for the survey. Two follow-up emails were sent at two-week intervals. Any local obstetric provider was eligible for the study. All participants were adults aged 18 and older. There were no other criteria for eligibility. There was no financial incentive offered for participation. Completion of the survey implied informed consent. Study procedures were approved by the Institutional Review Board at the University of Rochester Medical Center.

Survey data were collected and managed using REDCap electronic data capture tools hosted at the University of Rochester Medical Center. A definition of the PPV was provided for all respondents after initially asking about whether they had heard about the PPV. There were multiple-choice questions with open-ended responses available for subjects who selected “other” as a response to a multiple-choice question. Respondents were also given the option to provide additional free-text responses at the end of the survey.

This study applied simple descriptive statistics and chi-square analysis to assess survey responses by type of practice, type of provider, number of years practicing, and number of deliveries per month. Denominators used in calculating frequency distributions may vary as we excluded providers not responding to a particular question for the purposes of analysis, unless otherwise indicated. Percentages may not add up to 100% for questions asking respondents to “check all that apply”. Responses to open-ended questions were categorized by knowledge, attitudes, and practices according to survey question structure.

## Results

Of the 304 individuals eligible for the study, 90 completed the survey. Respondents reflected a diverse population of obstetric providers in Rochester, NY. Providers practiced in both private and public/teaching settings; ranged in their training as a birthing provider, including advanced practice providers, midwives, and physicians (obstetricians, family medicine physicians); and were from across all racial/ethnic groups (Table 1).

**Table 1:** Survey participant demographic and practice characteristics

Characteristics	n	%
Race		
American Indian or Alaska Native	3	3
Asian	8	9
Black or African American	1	1
White	72	80
Other	3	3
Prefer not to answer	3	3
Ethnicity		
Hispanic or Latinx	4	5
Not Hispanic or Latinx	79	91
Prefer not to answer	4	5
Gender		
Female	71	80
Male	16	18
Prefer not to answer	2	2
Type of Practice		
Private	20	23
Public/university	64	73
Other	4	5
Type of provider		
OBGYN	61	69
Family Medicine Physician	2	2
Midwife	19	21
Nurse Practitioner	2	2
Nurse	2	2
Physician Assistant	3	3
Stage in training (if physician)		
Resident	15	25
Fellow	4	7
Attending	42	69
Years of practice experience (excluding training)		
< 5 years	30	34
5–9 years	15	17
10–19 years	22	25
20–29 years	10	11
> 30 years	11	13
Deliveries per month		
< 10	21	24
11–20	42	48
21–30	9	10
31–40	1	1
> 40	14	16

Table 2 outlines respondents’ answers to questions about their knowledge, attitudes, and practices regarding the PPV. Sixty-six respondents (73%) said they had heard of the PPV (Table 2). Of the 66 respondents that had heard of a PPV, 45 said they referred their patients for a PPV. Providers that referred their pregnant patients to a pediatrician varied in their practices. Four of these providers noted that they refer patients who are interested and who ask about a pediatrician. Eighty-three respondents (95%) either agreed or strongly agreed that expectant parents should find a pediatrician before a baby is born.

**Table 2:** Knowledge, attitudes, and practices regarding the pediatric prenatal visit

Survey Question	n	%
Expectant parents should find a pediatrician before a baby is born		
Strongly agree	56	64
Agree	27	31
Neutral	3	3
Disagree	1	1
Strongly disagree	0	0
Have you heard of the “pediatric prenatal visit”?		
Yes	66	73
No	22	24
Don’t know	2	2
Have you referred your patients for a pediatric prenatal visit before? (n = 90)		
Yes	45	50
No	22	24
Don’t know	1	1
If yes, which of your patients do you refer for a pediatric prenatal visit?		
All pregnant patients	27	54
High-risk patients	2	4
First-time pregnant patients	15	30
Low-income pregnant patients	2	4
Other	4	8
What barriers are there to referral of your patients for a pediatric prenatal visit? (n = 63)		
Busy practice	20	32
Parent reluctance	18	20
Finances	10	16
Patient lack of resources	26	41
Patient lack of knowledge	31	49
Other	16	25

We sorted open-ended responses by categories of knowledge, attitudes, and practices.

### Knowledge and Understanding of the PPV

“My assumption is that the PPV is a specified process, and not just telling a patient, ‘You should look into picking a pediatrician before you deliver.’ I ask because, just as the occasional woman “shops” for her OB, this might be the case as well for pediatricians. If it IS indeed an actual program, I was not aware of it *per se*, so have not referred any patients to it.”

“My own lack of knowledge about the importance of this visit to the pediatrician—i.e. do they want this kind of visit or is it just an obligation and they…are just as happy to meet the family when the baby is born.”

“I am not sure whether the pediatric prenatal visits are reimbursed for the pediatric office. If they run a busy office, they may not have the capacity to see these ‘meet and greet’ visits.”

“Unsure where to refer.”

“I didn’t know this was a recommendation, but it makes perfect sense to me.”

“I only know about this practice due to the pediatrician I selected for my own child, and their recommendation for a pre-birth visit.”

### Attitudes About the PPV

“This would be a great idea. I am an OB resident and we don’t interact much with the pediatric side. It would be nice if we could arrange this to be done at OB visits.”

“I think it’s a wonderful idea to help set up our patients for success.”

“I did one with my [partner] when we were pregnant the first time around. It was very helpful for [them] as [they are] a planner.”

### Practices/Barriers Related to Facilitating PPVs for Their Patients

“We give them a list of practices and recommend they call to make an appointment. I don’t actually send a referral.”

“A lot of my patients are not educated on the subject and so don’t even know they have to pick a doctor for the baby before it’s born (like maybe they think the OBGYN will be the baby’s doctor too).”

“[Patients] do not have the time.”

“Other pregnancy medical conditions to address.”

“Patients typically find a pediatric provider without a referral.”

“I’ve had them call the pediatrician’s office only for them to be told they do not need one.”

Referral behaviors differed significantly by level of training and experience: attending physicians were more likely to refer compared with residents and fellows, and providers with more than ten years of experience were more likely to refer than providers with less than ten years of experience (Table 3). Referral behaviors did not differ by the type of practice, number of deliveries per month, or type of provider.

**Table 3:** Number of respondents by characteristic who referred patients to a pediatric prenatal visit

Characteristic	Referral	No Referral	p (Chi square test)
Stage in Training			< 0.001
Resident/fellow	1	18	
Attending	27	15	
Years of Experience			< 0.001
< 10 years	1	3	
10 + years	31	12	

## Discussion

It appears that utilization of the PPV has been very low for decades. Our results in this pilot study indicate little improvement despite passage of the ACA over a decade ago and updated guidelines from the AAP and ACOG in 2017. Although three-quarters of our respondents reported knowing about the PPV, the open-ended comments revealed confusion about the nature of the visit; for example, some equated it with “doctor shopping.” This suggests that basic knowledge about the PPV among obstetric providers remains low. Some providers who did correctly describe the PPV recognized that their knowledge was based on personal experience rather than professional training. We would expect higher referral rates in providers at earlier stages of their career because of expected education surrounding the relatively new guidelines from 2017; however, we observed the opposite trend. This suggests no systematic dissemination of these guidelines is occurring. This knowledge gap evidently presents an opportunity for educating obstetric providers about the PPV, especially given their positive attitudes toward this visit.

Although providers reported that the biggest barrier to referral is the patients’ lack of awareness about the PPV, some providers wrote about only referring for a PPV when the patient asks for one. To reduce barriers to delivering advice to patients about obtaining a PPV, provider education should cover not only the rationale for the visit, but also practical aspects of facilitating utilization—especially for low-income and first-time parents. Graduate medical education could serve as an appropriate time to educate trainees about the importance of the PPV and its accessibility. This study uses the framework of knowledge, attitudes, and practices. Knowledge is foundational for medical guideline adherence and is usually easier to achieve than changing attitudes, skills, and behaviors (de Leeuw et al., 2019). Our findings suggest a widespread lack of knowledge about the PPV among providers of obstetric care. This is useful for considering potential interventions to enhance guideline adherence. Knowledge alone is frequently insufficient for changing behavior; there are numerous barriers to implementation of physician practice guidelines including time limitations and lack of a reminder system, as well as the inertia of previous practice (Cabana et al., 1999). Nevertheless, when knowledge is very low, and the desired behavior change is perceived as simple and valuable, then dissemination of knowledge can change physician behavior. (Aligne et al., 2020). Future studies could determine the feasibility, acceptability, and impact of introducing educational interventions for medical professionals about the PPV.

A limitation of employing the knowledge/attitudes/practice framework for investigating guideline adherence is that we were looking for answers related to medical education and practice, but survey responses fell outside that scope. We were surprised to find, for example, that respondents’ knowledge of the PPV was sometimes related only to their personal experiences as parents. Future studies could perform deeper thematic analyses and delve into a greater variety of human factors influencing use of the PPV.

While this study was limited to obstetric providers in Rochester, NY, we have no reason to believe local providers would differ greatly in knowledge, attitudes, and practices from those in another geographic region. Future research could revisit this topic in a national survey of obstetric providers including advanced practice providers, midwives, obstetricians, and family medicine physicians. Including pediatric care providers (including pediatricians, family medicine physicians, and advance practice providers) could also be beneficial. Pediatricians could play an important role in promoting the PPV through advocating for these visits in their practice and with existing families. The AAP states that even non-first-time parents can benefit from the PPV to discuss how children can adapt to a newborn (Yogman et al., 2018). A survey of parents would be important for planning community interventions, e.g. public awareness campaigns. A local intervention to promote the PPV can be piloted to determine its feasibility and acceptability before transitioning to a larger scale.

The literature on this topic is limited and somewhat dated, with key studies on the benefit of the PPV having been published in the early 1980s. Many things have changed since then, and it is possible that such visits may be more or less useful now, or that their benefits could be achieved without an in-person visit to a doctor, e.g. via telemedicine. It would thus be valuable to explore the current utility of the PPV beyond this small pilot survey.

A strength of this survey was the inclusion of open-ended questions, which uncovered providers’ confusion about the PPV. Without these open-ended questions, we would have been led to believe that most providers are aware of and do refer their patients to a PPV. With these descriptive responses, we identified a knowledge gap.

The 30% response rate to our survey may indicate a general lack of interest in this topic. If that is the case, then the sample would be biased toward those who have greater familiarity with the PPV, and a more complete survey would find even lower knowledge about the PPV than indicated here.

## Conclusions

These findings suggest that a major barrier to routine adherence with the AAP and ACOG guidelines recommending the pediatric prenatal visit is a lack of familiarity with this type of service. The pediatric prenatal visit is not merely a “meet and greet” visit; it is a recognized component of comprehensive prenatal care. Disseminating information about existing evidence-based guidelines to patients and providers could increase awareness of the PPV, reduce disparities in its utilization, and improve health outcomes for mothers and children.

## Data Availability

Not applicable.
